# The heart in congenital diaphragmatic hernia: Knowns, unknowns, and future priorities

**DOI:** 10.3389/fped.2022.890422

**Published:** 2022-08-16

**Authors:** Neil Patel, Anna C. Massolo, Ulrike S. Kraemer, Florian Kipfmueller

**Affiliations:** ^1^Department of Neonatology, Royal Hospital for Children, Glasgow, United Kingdom; ^2^Ospedale San Filippo Neri, Rome, Italy; ^3^Intensive Care Unit, Department of Pediatric Surgery, Erasmus MC-Sophia Children’s Hospital, Rotterdam, Netherlands; ^4^Department of Neonatology and Pediatric Intensive Care, Children’s Hospital, University of Bonn, Bonn, Germany

**Keywords:** congenital diaphragmatic hernia, ventricular function, ventricular hypoplasia, pulmonary hypertension, echocardiography, biomarkers, cardiac

## Abstract

There is growing recognition that the heart is a key contributor to the pathophysiology of congenital diaphragmatic hernia (CDH), in conjunction with developmental abnormalities of the lung and pulmonary vasculature. Investigations to date have demonstrated altered fetal cardiac morphology, notably relative hypoplasia of the fetal left heart, as well as early postnatal right and left ventricular dysfunction which appears to be independently associated with adverse outcomes. However, many more unknowns remain, not least an understanding of the genetic and cellular basis for cardiac dysplasia and dysfunction in CDH, the relationship between fetal, postnatal and long-term cardiac function, and the impact on other parts of the body especially the developing brain. Consensus on how to measure and classify cardiac function and pulmonary hypertension in CDH is also required, potentially using both non-invasive imaging and biomarkers. This may allow routine assessment of the relative contribution of cardiac dysfunction to individual patient pathophysiological phenotype and enable better, individualized therapeutic strategies incorporating targeted use of fetal therapies, cardiac pharmacotherapies, and extra-corporeal membrane oxygenation (ECMO). Collaborative, multi-model approaches are now required to explore these unknowns and fully appreciate the role of the heart in CDH.

## Introduction

The past decade has seen growing appreciation of the heart as a key component of disease pathophysiology in congenital diaphragmatic hernia (CDH) (1). The application of advanced imaging modalities combined with multi-center registry analysis has shone new light on fetal cardiac development and the role of postnatal cardiac function, alongside pulmonary hypertension and pulmonary hypoplasia, in determining clinical phenotypes and outcomes in CDH. However, there is much more to understand in relation to cardiac development, mechanisms of dysfunction, and their clinical significance throughout life. Addressing these uncertainties may lead to new therapeutic strategies and improved outcomes in CDH.

This review aims to provide a current “state of the art,” comprehensively reviewing what is known of the heart from fetal life to adulthood in CDH, highlighting the major areas of ongoing uncertainty, and identifying key priorities for further investigation.

## The fetal heart in congenital diaphragmatic hernia

Cardiac abnormalities in CDH, as with those in the lungs and pulmonary circulation, undoubtably have their origins in the fetus.

### Congenital heart disease in fetal congenital diaphragmatic hernia

Congenital diaphragmatic hernia and congenital heart disease (CHD) are frequently associated. In recent systematic review up to 15% of live born CDH patients also have CHD, though rates may be as high as 28% when stillborn and terminated CDH cases are included (2). Conversely, 0.3% of infants requiring surgery for CHD have associated CDH (3).

Forty-two percent of infants with CDH and CHD are considered to have critical lesions, rather than simple shunts (ventricular and atrial septal defects, patent ductus arteriosus). The commonest associated cardiac lesions are, in order of frequency, ventricular and atrial septal defects, hypoplastic left heart syndrome (HLHS), coarctation of the aorta or aortic hypoplasia, tetralogy of Fallot and double outlet right ventricle (2, 4).

The relationship between measures of CDH severity (e.g., defect size, abdominal organ position, fetal lung size) and CHD incidence is as not yet understood. Similarly, the potential shared mechanisms, including genetic and environmental factors, contributing to CDH and associated CHD remain unclear, but may involve disruption of common pathways in cardiac and diaphragmatic development (5, 6). An increasing number of genetic mutations have been identified in CDH, however, the frequency of these is not affected by the presence or absence of associated anomalies including CHD (7, 8). Of note, HLHS and aortic anomalies may represent one end of a spectrum of left heart hypoplasia in CDH that is distinct from other mechanisms of CHD, as discussed in more detail below.

The presence of any CHD significantly affects surgical management of the diaphragmatic hernia. Systematic review indicates that CDH repair rates are lower (72% vs. 85%), patch repair more frequent (45% vs. 30%), and minimally invasive approaches are employed less often (5% vs. 17%). CDH repair typically precedes any cardiac intervention, and notably only 10% of affected cases received a cardiac intervention during the neonatal period (2). ECMO use is similar between CHD and non-CHD groups (9). The commonest cardiac surgeries performed in CDH cases are hybrid procedures, coarctation and aortic arch repair, VSD repair and pulmonary artery banding (3).

Importantly, any CHD confers lower survival rates in CDH, which approach 50% overall and as low as 30% for infants with critical cardiac lesions, and 1–5% for infants with CDH and HLHS (2, 3, 9, 10). Conversely, the presence of CDH also confers higher overall and peri-operative mortality when compared to all CHD, as well as increased rates of post-op complications and longer length of stay after cardiac surgery in both high and low risk cardiac lesions (3, 4).

Though no formal guidelines exist for management of CHD in CDH, an algorithmic, team approach has been advocated to assist in complex decision-making for critical lesions (11).

The remainder of this review will now focus on abnormalities of cardiac structure and function distinct from classical CHD, and which appear to be specific to CDH pathophysiology.

### Cardiac hypoplasia in congenital diaphragmatic hernia

Hypoplasia of the developing heart in CDH is an established finding, observed first in post-mortem studies and confirmed by echocardiography analyses, Table 1 (12–14).

**TABLE 1:** Echocardiographic studies of fetal and early postnatal cardiac dimensions and fetal cardiac function in CDH.

References	*N*	Gestation/postnatal age	Fetal heart dimensions	Neonatal dimensions	Outcome
Schwartz et al. (33)	20 L CDH	“On [neonatal] admission”	–	Lower LV mass in CDH vs. controls	LV mass lower in cases who required ECMO
Thebaud et al. (19)	40 fetal 32 newborn CDH	21–30 and 31–40 weeks	Reduced LV:RV (MV:TV and Ao:PA) ratios at 31–40 weeks	–	LV:RV at 31–40 weeks correlated with non-survival and PH.
Baumgart et al. (16)	23 newborn CDH	38–40 weeks	–	Reduced Ao, LV mass, MV diameter and increased PV diameter in CDH.	LV mass lower in non-survivors
VanderWall et al. (17)	12 CDH fetus	17–25 weeks	Reduced RV (TV) and LV (MV) width, LV volume and mass in CDH.	–	No difference in fetal dimensions in survivors vs. non-survivors
Van Mieghem et al. (27)	27 fetal L CDH, 117 controls	–	LV ED dia smaller in CDH. No difference in LV function (EF, FS, MPI) in CDH vs. controls.	–	FETO did not affect cardiac size but reduced MPI. Reversal of FETO did not affect cardiac size or function.
Stressig et al. (24)	32 CDH fetuses	19–39 weeks	Reduced *z*-score of MV, Ao valve, MV:TV, Ao/PA in cases with ductus venosus and IVC streaming to right heart	–	
Vogel et al. (15)	125 111 L CDH 14 R CDH	24 (17–39) weeks	Age-adjusted AV, MV, LV length, LV volume, were all smaller in CDH	*Z*-scores of left heart structures increased from prenatal to postnatal echo	No association between prenatal left heart *Z*-scores and postnatal survival
DeKoninck et al. (21)	17 R CDH fetus, 17 controls	27 (24–29) weeks	Reduced PV, RV ED and RV ES diameters, RVO and RV SV in CDH. No difference in AoV and LV dimensions or MPI.	–	–
DeKoninck et al. (151)	38 fetuses, 29 L CDH 9 R CDH	27 (21–32) weeks	Increased LV strain in CDH, no correlation with O:E LHR	–	–
Yamoto et al. (32)	99 controls, 33 CDH fetus	Control 32 (17–39) CDH 32 (21–40) weeks	Cardiothoracic area (CTAR) ratio, MPA:Ao, TV:MV all s altered in CDH, before and after 32 weeks gestation	–	CTAR, MPA:Ao and TV:MV all differentiated survivors vs. non-survivors. TV:MV had greatest sensitivity
Byrne et al. (14)	188 fetuses, 171 L CDH, 17 R CDH	16–37 weeks	MV, AV, LV volume and LVO, reduced in “severe CDH” (LHR < 1 and liver in chest in L CDH).	–	
Degenhardt et al. (25)	8 CDH fetus pre and post FETO		No significant change in function (TAPSE, MAPSE, MPI) pre and post FETO	–	–
Kailin et al. (31)	52 L CDH fetus	27 ± 5 weeks and earliest postnatal echo	–	AV and LV SAX dimension *z*-scores significantly lower prenatally vs. postnatally	Fetal AV *z*-score independently associated with iNO use
Lemini et al. (28)	31 L CDH fetus, 75 controls	34 ± 6 weeks	Impaired diastolic function in fetal CDH assessed by tissue Doppler imaging.	–	–
Kaya et al. (29)	28 CDH 20 L CDH 8 R CDH. 56 controls		RV parameters only. No difference in RV TDI velocities. Increased ICT, IRT and RV MPI in CDH	–	–
Coffman et al. (30)	52 infants, 40 L CDH, 12 R CDH	Birth – 1 month of age	–	Reduced *z*-scores for LVIDd, LVIDs, aortic annulus, arch, sino-tubular junction	Length of stay inversely correlated with left heart structures
Massolo et al. (18)	12 L CDH fetus, 41 controls	24–26 weeks, 30–32 weeks, 34–36 weeks	Reduced MV, LV area, TV and RV area, MV:TV at 24–26 weeks. At 34–36 weeks reduced MV, LV area, and MV:TV.	–	MV and MV *z*-score at 24-26 weeks associated with death/ECMO

LV, left ventricle; RV, right ventricle; MV, mitral valve; TV, tricuspid valve; Ao, aortic diameter; PA, pulmonary artery diameter; LVO, left ventricular output; EF, ejection fraction; FS, fractional shortening; MPI, myocardial performance index; FETO, fetal endoscopic tracheal occlusion; ED, end diastolic; ES, end systolic; SV, stroke volume; AV, aortic valve; SAX, short axis; TDI, tissue Doppler imaging; MPA, main pulmonary artery; ICT, isovolumic contraction time; IRT, isovolumic relaxation time; LVID, LV internal diameter.

In fetuses with left-sided CDH ventricular hypoplasia appears to predominantly affect the left ventricle (LV), characterized by reduced ventricular width and associated reductions in LV area and mass, together with reduced aortic valve diameter (14, 15). Right ventricular (RV) dimensions may also be reduced at earlier gestation but appear to increase, along with pulmonary artery diameter, at later gestation (16, 17). Accordingly, ratios of left to right cardiac dimensions in left-sided CDH are lowest at later gestations (18–20). Conversely, in right-sided CDH the limited available data indicate reduced fetal right ventricular and pulmonary arterial dimensions combined and less severe LV hypoplasia than in left-sided CDH (14, 21).

**FIGURE 1:**
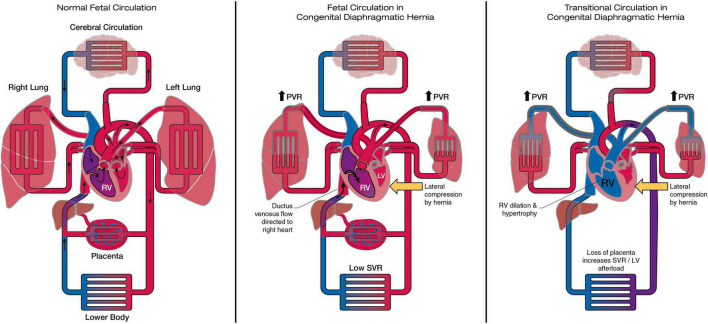
The heart and circulation in CDH during the fetal and transitional period. In the fetal CDH circulation LV hypoplasia may be related to redirection of ductus venosus flow to the right heart and reduced pulmonary blood flow, together with lateral compression by the herniating abdominal contents. In the transitional period removal of the placenta increases afterload on the ventricles. The right ventricle dilates and becomes dysfunctional in the face of sustained postnatal increase in PVR. LV function is at risk due to pre-existent hypoplasia, septal displacement and the acute increase in afterload. RV, right ventricle; LV, left ventricle; PVR, pulmonary vascular resistance; SVR, systemic vascular resistance.

Multiple mechanisms of fetal cardiac hypoplasia have been proposed, though the relative contribution and timing of these remains uncertain (Figure 1):

1. Mechanical compression of the developing heart by herniating abdominal contents. Consistent with this hypothesis ventricular hypoplasia is greatest on the ipsilateral side and appears to disproportionately affects ventricular width rather than length (14, 18). Analogous reductions in fetal mitral valve and AV diameter are observed in fetuses with large, compressive left-sided congenital lung masses (22).
2. Reduced pulmonary blood flow in CDH compared to normal fetuses, due to the primary structural changes in the pulmonary vasculature characteristic of CDH. This in turn leads to reduced pulmonary venous return, LV filling and growth. In support of this flow-based mechanism reduced LV dimensions are similarly observed in fetuses with anomalous pulmonary venous drainage (23). This mechanism would in theory have greatest impact later in gestation when, in the normal fetus, increases in fetal pulmonary blood flow and LV size are usually observed.
3. An additional flow-based mechanism of LV hypoplasia in CDH may be due to mediastinal shift and liver herniation resulting in re-direction of ductus venosus (DV) and inferior vena cava (IVC) streaming away from the foramen ovale and resulting in reduced LV filling and growth (24). Oxygenated venous return is instead re-directed to the right heart and thereafter the majority will pass across the patent ductus and a minority to the pulmonary arteries. Increased oxygenation of the fetal pulmonary blood flow, as a secondary consequence of this mechanism, has also been hypothesized as a possible cause of the excessive muscularization of the pulmonary arterioles in CDH.

### Fetal cardiac function in congenital diaphragmatic hernia

Prenatal cohort studies of myocardial function have not to date demonstrated significant fetal cardiac dysfunction (25–29). Theoretically, the fetal circulation may mitigate against cardiac dysfunction *in utero*; the presence of a patent ductus and low resistance placenta protecting the RV from excessive afterload (despite increased resistance in the pulmonary vasculature) and ensure that the non-dominant, hypoplastic LV remains untaxed by excessive preload or afterload, Figure 1. Prospective studies throughout gestation are needed to understand the natural history and clinical significance of fetal cardiac function.

### Clinical significance of fetal cardiac hypoplasia in congenital diaphragmatic hernia

The relationship between fetal cardiac hypoplasia and clinical outcome remains unresolved. In single center cohorts of left-sided CDH left heart dimensions, notably LV width, mitral and aortic valve diameters, and ratios of left to right-sided dimensions have been associated with adverse outcome including higher neonatal mortality, higher ECMO use, increased inhaled nitric oxide use, and prolonged length of stay (18, 19, 30–33).

However, these findings have not been replicated in other series. VanderWall did not observe any association between fetal ventricular dimensions and outcome, though small cohort size may have been a factor (17). Vogel et al. observed mild to moderate fetal LV hypoplasia in a cohort of 125 CDH cases. LV fetal dimensions, expressed as *z*-scores, were not associated with postnatal survival when analyzed as continuous data, but were on categorical analysis (15). In the same cohort, there was a trend toward normalization of LV dimensions on paired postnatal, post-CDH repair, echocardiograms. This may suggest that reduced LV volumes are recruitable in response to postnatal hemodynamics.

Further investigation is a priority to determine which, if any, fetal cardiac dimensions have the greatest prognostic utility, when and how these should be measured, and the mechanisms by which these might directly influence postnatal LV dimensions, function, and outcome.

### Cellular and metabolic function in the fetal heart in congenital diaphragmatic hernia

Whether structural changes in the fetal heart in CDH are associated with primary or secondary abnormalities at a genetic, epigenetic, cellular or metabolic level remains largely unknown. Cardiac hypoplasia in nitrofen rat models of CDH is associated with reduced expression of insulin-like growth factor-1, epidermal growth factor, basic fibroblast growth factor and platelet derived growth factor, Table 2 (34, 35). Zhaourigetu et al. also recently observed abnormal cardiomyocyte structure associated with reduced expression of mitochondrial and fatty acid biogenesis genes in this CDH model (36).

**TABLE 2:** Studies of fetal cardiac cellular structure and metabolism in CDH.

References	Experimental CDH model	Findings
Karamanoukian et al. (38)	Fetal lamb	No difference in ventricular wall thickness, total protein, DNA collagen, and elastin between CDH and controls
Tannuri (39)	Fetal rabbit	Decreased ventricular wall thickness, increased septal thickness.
Teramoto and Puri (34)	Nitrofen rat	Decreased insulin like growth factor-1 (IGF-1) and epidermal growth factor (EGF) expression in CDH hearts associated with cardiac hypoplasia.
Guarino et al. (35)	Nitrofen rat	Expression of basic fibroblast growth factor (bFGF) and platelet-derived growth factor (PDGF) was significantly reduced in CDH heart, with associated reduced heart growth.
Baptista et al. (152)	Nitrofen rat	Significant oscillation in BNP and angiotensin mRNA in nitrofen exposed pups compared to controls, but not in CDH specifically.
Pelizzo et al. (37)	Post mortem 7 human CDH fetuses	Dis-homogenous growth factor distribution in ventricles in fetal CDH. Increased small intramyocardial artery density and increased vascular thickness in ventricular walls.
Zambaiti et al. (153)	Fetal lamb	Early tracheal occlusion was associated with LV myocardial enlargement, increased endothelin-1 (ET-1) and transforming growth factor beta (TGF beta) expression.
Zhaorigetu et al. (36)	Nitrofen rat	Increased ventricular myocyte hypoxia, downregulation of mitochondrial and fatty acid biogenesis genes. Altered mitochondrial structure.

A single post-mortem study of hearts from human CDH fetuses also demonstrated dis-homogenous growth factor expression (37). In the same study fetal CDH hearts had abnormal thickening and proliferation of intra-myocardial vessels, particularly in the inter-ventricular septum. This raises the hypothesis that intra-cardiac vasculature in CDH might demonstrate developmental abnormalities analogous to those in the pulmonary vasculature (18).

In fetal rabbit models of CDH, though not lamb models, cardiac hypoplasia is associated with decreased ventricular wall thickness and increased septal thickness (38, 39). Ventricular wall thickening, due to cardiomyocyte hyperplasia, is also observed in hypoplastic left heart syndrome (HLHS), potentially pointing to common mechanisms of cardiac dysgenesis in these two conditions (40).

### Fetal therapies and the developing heart in congenital diaphragmatic hernia

In limited cohort studies fetal tracheal occlusion (FETO), the principal prenatal therapy attempted for CDH, does not appear to significantly affect fetal cardiac dimensions or function in CDH (16, 18). Of note, the recent larger multicenter TOTAL trials of FETO did not include assessment of cardiac dimensions or function FETO (41).

A variety of pre-natal pharmacotherapies aimed at modulating pulmonary vascular development have been investigated in pre-clinical CDH models (42). Sildenafil, a phosphodiesterase 5 inhibitor partially reverses pulmonary vascular abnormalities and lowering pulmonary vascular resistance and reducing RV hypertrophy in animal models (43–45). However, adverse events associated with of prenatal administration in non-CDH patients have prevented clinical trials in diaphragmatic hernia (46). Maternal hyperoxygenation may be an alternative therapeutic approach which in congenital heart disease has been observed to increase pulmonary blood flow, and could potentially increase left ventricular flow and size in turn (47, 48). Further investigation is required of the impact of prenatal therapies on cardiac development and postnatal function in CDH.

## The heart in early postnatal life in congenital diaphragmatic hernia

Changes in the fetal heart in CDH may be important precedents of postnatal cardiac function. Recent investigation using functional echocardiography and multi-center registry analysis have led to new, but still incomplete, understanding of the heart’s unsteady transition from pre-natal to post-natal environment in CDH, Table 3. Figure 1 provides a visual overview of these changes in loading conditions, cardiac morphology and function from fetal to postnatal life.

**TABLE 3:** Early postnatal cardiac function in CDH.

References	Population	Parameter	Ventricular function and relationship to outcome
Patel et al. (54)	9 CDH, 28 controls	RV MPI	Reduced RV MPI in CDH
Patel et al. (53)	11 CDH infants median 18 days. 28 controls.	TDI myocardial velocities and TV Doppler velocities	Reduced RV early diastolic velocities in CDH.
Aggarwal et al. (154)	29 CDH, 27 controls. <3 days	Systolic:Diastolic time durations	Reduced RV diastolic time intervals in CDH, and in CDH non-survivors.
Aggarwal et al. (52)	34 CDH, 35 controls	RV and LV MPI and cardiac index (CI)	Reduced RV and LV MPI, and CI in CDH compared to controls, and CDH cases who died/required ECMO. LV MPI and CI associated with mortality.
Moenkemeyer and Patel (49)	16 CDH infants (13 L CDH, 3 R CDH) day 1–2	TDI myocardial velocities	Reduced RV early diastolic myocardial velocities in non-survivors. RV diastolic dysfunction correlated with increased length of stay and duration of respiratory support
Altit et al. (60)	34 CDH, first 48 h.	STE-derived strain. RV FAC and TAPSE. EF.	Reduced RV and LV longitudinal strain and strain rate, RV TAPSE and FAC, and LV EF in CDH cases who required ECMO.
Patel et al. (63)	25 CDH (21 L CDH) and 20 controls in first 48 h of life	TDI and STE-derived strain	Global reduction in RV and LV systolic strain in CDH. LV longitudinal strain correlated with fetal lung volume, duration of intubation and length of stay, and was lower in non-survivors/ECMO.
Altit et al. (55)	44 CDH, 18 controls. First 48 h	Ventricular strain. RV FAC, TAPSE. LV EF, stroke distance	Reduced RV and LV longitudinal strain, reduced RV FAC and TAPSE, and LV stroke distance in CDH.
Naguib et al. (56)	20 CDH infants	RV outflow VTI	Lower RV output in CDH non-survivors.
Gaffar et al. (66)	27 CDH cases (21 L CDH)	RV and LV CI and VTI, LV EF	Lower LV CI in CDH cases who received ECMO.
Patel et al. (62)	1173 CDH infants, (971 L, 202 R). First 48 h of life	CDH Registry analysis. Cardiac function reported by 59 centers	Cardiac function normal in 61%, RV dysfunction in 15%, LV dysfunction in 5%, biventricular dysfunction in 19%. LV and biventricular dysfunction associated with increased mortality. RV and LV dysfunction associated with ECMO
Avitabile et al. (61)	220 CDH (184 L CDH).	RV strain, FAC, FWS pre-op, post op (<1 week) and recovery phase (>1 week)	Abnormal RV strain associated with ECMO use. Abnormal RV strain in recovery phase associated with increased mortality. Improvement in net RV strain after repair.

RV, right ventricle; MPI, myocardial performance index; TDI, tissue Doppler imaging; TV, tricuspid valve; CI, cardiac index; STE, speckle tracking echocardiography; FAC, fractional area change; TAPSE, tricuspid annular plane systolic excursion; EF, ejection fraction; VTI, velocity-time integer; EF, ejection fraction; FWS, fractional wall shortening.

### Right ventricle dysfunction in congenital diaphragmatic hernia

Dysfunction in the RV, in conjunction with ventricular dilatation and hypertrophy, has been demonstrated from the first days of life in CDH (49). These are considered to result from pathological increases in RV afterload as a result of the structural and functional pulmonary vascular abnormalities characteristic of CDH (50). Increased RV pressure, and a concomitant reduced systemic pressure, may also reduce coronary artery flow gradient, leading to RV ischemia and dysfunction. Whilst recognized in other pulmonary hypertensive disease this mechanism has not been studied directly in CDH (51).

Early RV dysfunction in CDH is characterized by both impaired systolic function and, notably early diastolic dysfunction, demonstrated by reduced diastolic myocardial velocities and shortened diastolic duration in the RV (52–55).

Right ventricle dysfunction, when present, likely contributes to early clinical instability *via* a number of mechanisms. First, the failing RV may become “uncoupled” from the pulmonary circulation, unable to maintain adequate pulmonary blood flow, contributing to impaired oxygenation, and reduced LV filling and output (56). Second, RV dysfunction negatively impacts LV performance *via* mechanisms of ventricular interdependence including shared myocardial fibers, disruption of normal systolic and diastolic time intervals, and septal displacement reducing LV volume (40). Through these mechanisms RV dysfunction may be a key mediator of adverse effects of pulmonary hypertension in CDH (57, 58).

In cohort studies early RV dysfunction is associated with prolonged duration of respiratory support, increased mortality and ECMO use (49, 59–61). In a recent large multi-center registry analysis of cardiac function in the first 48 h of life RV dysfunction was present in 34% of CDH cases either in isolation in combination with LV dysfunction, and was associated with increased mortality and ECMO use (62).

### Left ventricular dysfunction in congenital diaphragmatic hernia

The potential for postnatal left ventricular dysfunction in newborns with CDH has been long-recognized by experienced clinicians, and more recently quantified using functional echocardiographic techniques (52, 55, 63). Its importance as a component of CDH pathophysiology was highlighted by recent multi-center analysis demonstrating that LV dysfunction, in isolation or combined with RV dysfunction, independently predicted death and ECMO use (62).

Early postnatal LV dysfunction is frequent. In registry analysis of over 1100 CDH cases from 59 centers LV dysfunction was reported in 24% of CDH cases (62). However, this may be an underestimate; In smaller cohort studies utilizing sensitive, quantitative strain analysis of myocardial function LV dysfunction was observed in 56% of cases (63).

Postnatal LV dysfunction is characterized by both global systolic and diastolic dysfunction affecting longitudinal, circumferential and radial function, and dyssynchrony of myocardial segments (60, 63, 64). Postnatal LV volumes may also be reduced possibly due to compressive actions of a dilated RV and the herniated organs, combined with the legacy of fetal LV hypoplasia.

Left ventricle dysfunction is frequently observed in combination with RV dysfunction and may therefore be a secondary consequence *via* mechanisms of ventricular interdependence, as discussed above (64). However primary LV dysfunction may occur in the absence of, or disproportionate to, RV dysfunction (62). Multiple factors are hypothesized to contribute to primary postnatal LV dysfunction in the transitional period (1, 65):

I. Fetal LV dysfunction
II. Fetal LV hypoplasia
III. Changes in LV loading conditions: reduced preload, due to failure of normal increases in pulmonary blood flow at birth, and increased afterload due to removal of the low-resistance placenta from the systemic circulation
IV. Hypoxia and acidosis contributing to worsening ventricular function

Left ventricle dysfunction likely contributes to adverse clinical outcome *via* reduced LV output and systemic blood flow, resulting in impaired tissue oxygenation and a viscous cycle of worsening hypoxia and acidosis (66). Accordingly, the systemic hypotension frequently observed in early CDH is likely to be a consequence of impaired cardiac function and cardiac output, rather than hypovolemia or low systemic vascular resistance.

Early LV dysfunction is associated with other markers of CDH severity, including smaller fetal lung volumes, larger diaphragmatic defect size, and liver herniation (62, 63). However, Dao et al. have observed that LV dysfunction may also occur in “lower risk” CDH cases with smaller defects (67).

Of note, LV dysfunction appears to be a transitional phenomenon, apparently present from soon after birth but with the potential to improve rapidly over the first week of life (68, 69). This may have important consequences for individualized management strategies, including ECMO, as discussed below.

### Left ventricle dysfunction as a mechanism of pulmonary hypertension

Left ventricle dysfunction leading to increased end diastolic, left atrial and pulmonary venous pressures is well-recognized as a mechanism of increased pulmonary vascular resistance in adult heart disease (70). There is increasing recognition that similar mechanisms may occur in CDH in the setting of early, transition LV dysfunction, contributing to a post-capillary increase in pulmonary venous resistance, distinct from pre-capillary changes in pulmonary arterial resistance (1). This may have important implications for targeted management strategies in CDH, including the suitability of pulmonary vasodilators, as discussed below.

### Hemodynamic phenotypes in congenital diaphragmatic hernia

The complex interplay of ventriculo-arterial, inter-ventricular, and cardio-respiratory interactions in CDH results in dynamic hemodynamic phenotypes with variable RV and LV function and dysfunction. As discussed later, this concept may be important in targeted, individualized therapeutic strategies.

Atrial shunting patterns may help define these phenotypes in the clinical setting. As recently highlighted by Wehrmann et al., the frequent presence of left-to-right atrial shunting in CDH, despite elevated pulmonary artery pressures, should prompt a closer examination of the left ventricular size and function (71).

### Variability in cardiac dysfunction and clinical phenotypes

Current models of CDH are based on three key inter-related pathophysiologies; pulmonary hypertension, pulmonary hypoplasia, and cardiac dysfunction (65). However, the severity of each of these may be variable and disproportionate. Although severe cardiac dysfunction is associated with larger diaphragmatic defects, smaller lung volumes, and more severe pulmonary hypertension, it may also be observed in patients with smaller diaphragmatic defects and milder respiratory compromise (62, 63, 67). Improved recognition and characterization of individual clinical phenotypes may be an important concept in CDH, and potentially inform more effective, targeted therapies.

## Echocardiographic assessment of cardiac function and hemodynamics in congenital diaphragmatic hernia

The absence of standardized definitions and measurement tools are a major ongoing barrier to hemodynamic research and clinical management in CDH.

A variety of functional echocardiographic parameters have been used to assess cardiac function and pulmonary hypertension in CDH, as listed in Tables 1, 4. However, each has practical and technical limitations and there is no single “gold standard” measure (72). Though useful definitions of cardiac dysfunction and pulmonary hypertension have been employed in individual studies, there is no established consensus agreement on these (58, 73).

**TABLE 4:** Investigations of biomarkers of cardiac function and pulmonary hypertension in CDH.

References	Population	Plasma biomarker	Relationship to hemodynamic performance	Available for routine clinical use
Partridge et al. (84)	132 CDH	BNP	BNP correlated with pulmonary hypertension and need for ECMO. No cardiac function data.	Y
Guslits et al. (85)	49 CDH	BNP levels at age 1–5 weeks	BNP level predicted adverse outcome at 3–5 weeks (ongoing respiratory support or death). No cardiac function data.	Y
Avitabile et al. (61)	220 CDH	BNP levels pre-repair, post-repair and recovery (>1 week post repair)	Increased BNP level associated with reduced strain in recovery, but not pre- or immediately post-op.	Y
Baptista et al. (86)	28 CDH	NT-proBNP in first 24 h of life	NT-proBNP correlated with RV MPI, TV E:A, and PAP.	Y
Snoek et al. (89)	128 CDH	High sensitivity troponin (hsTnT) and NT-proBNP on day 1	NT-proBNP and hsTNT did not predict death, PH, ECMO, or BPD. No cardiac function data.	Y
Heindel et al. (87)	44 CDH	NT-proBNP at 6, 12, 24, and 48 h of life	NT-proBNP correlated with qualitative cardiac dysfunction at 24 h, 48 h, and 7 days, and was higher in ECMO group.	Y
Bo et al. (69)	63 CDH	NT-proBNP measured daily for the first 7 days on ECMO	Significantly higher NT-proBNP values on days 3–7 in patients with ECMO weaning failure. Doubling in mortality in patients with increasing NT-proBNP on days 4–7.	Y
Gupta et al. (88)	2337 CDH	NT-proBNP recorded during neonatal admission	NT-proBNP correlated with cardiac dysfunction (RV or LV), mortality and larger defects.	Y
Keller et al. (155)	40 CDH	Endothelin 1 (ET-1) measured serially in first 2 weeks of life	ET-1 correlated with PH at 2 weeks of age. No cardiac function data.	N
Patel et al. (90)	10 CDH	VEGFA and placental growth factor (PLGF) measured serially during neonatal period	VEGFA:PLGF ratio correlated with RV diastolic function, PH and oxygenation index, and higher in non-survivors at days 3 and 14.	N
Kipfmueller et al. (91)	30 CDH	Soluble receptor for advanced glycation end products (sRAGE) at 6, 12, 24, 48 h and 7–10 days	sRAGE lower in CDH than controls and lower in ECMO cases. sRAGE correlated with pulmonary hypertension and fetal lung volume. No cardiac function data.	N

BNP, brain natriuretic peptide; NT-proBNP, N terminal proBNP; RV, right ventricle; MPI, myocardial performance index; TV, tricuspid valve; PAP, pulmonary artery pressure; PH, pulmonary hypertension; BPD, bronchopulmonary dysplasia.

To enable consistent multi-center hemodynamic data collection four actions are required:

1. Recommendations for routine use of functional echocardiography in acute CDH care
2. International consensus on measurement parameters, definitions and classification of cardiac function and pulmonary hypertension based on existing international guidance and adapted specifically for CDH (74–78).
3. Exploration of multi-modal measures of hemodynamic performance in CDH, including systemic oxygen delivery, cardiac output and microcirculatory function.

## Bedside assessment of hemodynamic function in congenital diaphragmatic hernia

Echocardiographic assessment should be accompanied by wider multi-modal physiological assessment, as in any infant with hemodynamic instability. Where possible, near infrared spectroscopy enables monitoring of systemic oxygen delivery and may precede (79) changes in plasma lactate or end organ injury (80, 81). Exploratory studies are combining cerebral NIRS with EEG to investigate neuro-cardiovascular coupling (82).

Invasive arterial monitoring is a requisite during hemodynamic instability for monitoring of blood pressure and arterial gas sampling. However, line position and the impact of intra and extracardiac shunts must be considered. Right to left ductal shunting will decrease pH, PaO_2_ and increase PaCO_2_ of post-ductal measurements, whereas a right-to-left atrial shunt, whilst uncommon in CDH, will also reduce pre-ductal oxygenation (71, 83).

## Biomarkers of cardiac function in congenital diaphragmatic hernia

Plasma biomarkers may be useful in CDH to assess cardiac performance and pulmonary hypertension. These may be produced in response to hemodynamic compromise, or contribute to the primary pathways mediating CDH pathophysiology.

Natriuretic peptides brain natriuretic peptide (BNP) and its precursor N-terminal (NT) proBNP are established biomarkers in other pulmonary hypertensive diseases. BNP has a short half-life complicating measurement, but in CDH has been shown to be associated with pulmonary hypertension, need for ECMO, predictive of adverse outcome at 1 month of age, Table 4 (84, 85). Recently Avitabile et al. also demonstrated that BNP was associated with impaired RV strain after CDH repair, though not in the pre-operative period (61).

NT-proBNP has a longer half-life making clinical measurement more reliable, and has been observed to correlate with cardiac dysfunction, pulmonary hypertension, and ECMO use in case series and large registry analysis (86–88). However, neither NT-proBNP, nor high sensitivity troponin (hsTnT) correlated with outcomes in a recent RCT of ventilation modalities in CDH (89).

Other potential biomarkers include the vascular endothelial growth factor-A (VEGFA) and placental growth factor (PLGF), the ratio of which correlated with RV diastolic dysfunction in a pilot investigation (90). Also, the soluble receptor of advanced glycation end products (sRAGE) is a new potential mediator of endothelial dysfunction in CDH, associated with mortality, severity of PH, and adverse outcome in CDH (91). MicroRNA’s, a group of small non-coding RNA, are involved in the development and function of the lungs and the pulmonary vasculature in CDH, though to date their relationship to cardiac dysfunction in CDH remains unstudied (92–94).

## The heart and the brain in congenital diaphragmatic hernia

Congenital diaphragmatic hernia is associated with neurodevelopmental impairment in a significant minority of affected people (95). Altered fetal brain development and postnatal brain injury may be contributing factors, and neuro-cardiovascular interactions may be central to these (82, 96).

Abnormalities in ventricular size and function may plausibly lead to alterations in cerebral blood flow, as is also suspected in infants with hypoplastic left heart syndrome (97).

This is itself a major topic for further discussion and investigation in CDH, beyond the scope of this review.

## Management of cardiac dysfunction in congenital diaphragmatic hernia

### Physiological approaches to managing the transition at birth

Recent animal and human feasibility studies have explored the use of physiologically based strategies for managing the transition at birth, combining lung recruitment with delayed cord clamping. These have demonstrated short-term improvements in pulmonary blood flow, pulmonary artery pressure and systemic blood pressure, though the impact on cardiac function *per se* has not been directly investigated (98–100). Randomized trials are in progress, but do not include cardiac function as a key outcome measure (101, 102).

### Pharmacological therapies

Hemodynamic pharmacotherapy in CDH remains a challenging and unresolved issue. Historical approaches focused on maintaining systemic blood pressure and promoting pulmonary arterial vasodilation. The list of possible pharmacological therapies is ever-increasing, but with limited and often contradictory evidence leading to clinical confusion and risk of inappropriate use (103).

The current use of pulmonary vasodilators exemplifies this. Inhaled nitric oxide and sildenafil (in oral and intravenous formulations) are widely used in CDH patients with the intention of reducing pulmonary vascular resistance *via* endogenous nitric oxide pathways (104). However, only a minority of recipients demonstrate improved oxygenation, historic RCTs did not demonstrate improved outcomes, and recent registry analysis has suggested that iNO may be associated with increased mortality (105, 106).

Improved understanding of cardiac dysfunction may help address the uncertainty and anxiety around the use of these agents. The presence of LV dysfunction appears to be associated with non-response to iNO and sildenafil (107, 108). Possible mechanisms may be post-capillary hypertension secondary to LV dysfunction unresponsive to pre-capillary vasodilatation, or increased pulmonary blood flow exacerbating LV dysfunction (109).

The actions of other cardiovascular therapies which have been directly investigated in CDH are summarized in Table 5. Milrinone, a phospho-diesterase 3 inhibitor acting on the endogenous prostacyclin pathway appears well suited to treat both RV and LV dysfunction in CDH, for its inotropic, lusitropic and pulmonary vasodilating effects (110). In a case series of infants with pre-existent RV dysfunction milrinone use was associated with improved oxygenation and RV diastolic function (111). However, a recent retrospective analysis in mild to moderate CDH observed no effect on oxygenation or LV dimensions (112). An RCT of early milrinone use in CDH is ongoing but does not include cardiac dysfunction as an enrollment parameter (113).

**TABLE 5:** Hemodynamic therapies investigated in CDH.

Therapy	Class	Presumed actions	Use in CDH (62, 124, 127)	Evidence in CDH
Inhaled nitric oxide	Nitric oxide analog	Pulmonary vasodilator	62–65%	Improved oxygenation in minority (30%) of unselected recipients. No improvement in outcome (79). Non-response may be linked to LV dysfunction (81).
Sildenafil (IV or enteral)	Phospho-diesterase 5 inhibitor	Pulmonary and systemic vasodilator	IV: 16% Any: 22%	Improved oxygenation in minority of recipients. Non-response associated with LV dysfunction (64). Ongoing CoDiNOS RCT of IV sildenafil vs. iNO in progress (73).
Milrinone	Prostacyclin analog	+ve inotrope and lusitrope. Pulmonary and systemic vasodilator	33–42%	Improved oxygenation and RV diastolic velocities (84). No effect on LV dimensions and atrial and ductal shunts (85). RCT in progress (57).
Vasopressin	Vasopressin analog	Pulmonary vasodilator, systemic vasoconstriction	Not known	Increased blood pressure, reduced systemic:pulmonary artery pressure ratio, improved oxygenation (89).
Levosimendan	Calcium sensitizer	+ve inotrope	Not known	Improved RV and LV function and reduced vasopressor-inotrope score (88)
Prostaglandin E1	Prostaglandin	Maintain ductal patency, pulmonary vasodilator	9–11%	Improved indices of PAP, LV function and oxygenation (120–122).
ECMO	–	Mechanical support	50%	Improved biventricular function on ECMO (69)

Levosimendan a calcium-sensitizing drug is commonly used in infants with congenital heart defects in the setting of low cardiac output syndrome (114). There is preliminary evidence that levosimendan is also associated with improvement of right and left ventricular dysfunction and a decrease in the Vasopressor-Inotropic Score in CDH (115).

Vasopressin use in CDH has also been associated with improved blood pressure, and reduced pulmonary:systemic blood pressure ratio, though the specific impact on LV function remains unstudied (116). The utility of systemic vasoconstrictors in CDH is unclear, and may depend on individual patient pathophysiology. In the setting of severe LV dysfunction increasing afterload may exacerbate ventricular failure (117). However, if LV function is preserved and RV function impaired there may be theoretical benefits to increasing systemic vascular resistance; to improve RV coronary blood flow, augment LV function *via* the eponymous Anrep effect, and restore septal positioning (51, 118, 119). Further investigation is required to understand which patient phenotypes are likely to benefit, and which of these potential mechanisms are beneficial in the clinical setting.

Prostaglandin E1 (PGE_1_) use been proposed in early cardiovascular management in CDH to maintain ductal patency, as well as for its pulmonary vasodilating properties. In the setting of supra-systemic pulmonary hypertension a patent ductus permits right-to-left shunting, reducing the effective afterload on the RV, and supporting systemic blood flow, with theoretical benefits in the setting of both RV and/or LV dysfunction. Case series have demonstrated improvements in pulmonary artery pressure, oxygenation and LV function with PGE_1_ use, however the optimal timing, dosing and duration remains undefined (120–122).

From the investigations described here it has become clear that there is no universally effective single agent for treating cardiac function and pulmonary hypertension in CDH. However, that does not necessarily mean that current therapies are ineffective. Instead new therapeutic *strategies* may be required, based on characterization of individual patient phenotype, including the relative contributions of RV and LV dysfunction, pre and postcapillary PVR, and ductal patency. This will allow investigation of the efficacy of targeted, pathophysiology-based therapeutic approaches, rather than indiscriminate use of single agents.

Hydrocortisone is also frequently used in the management of infants with CDH as an adjunct treatment for hypotensive cardiovascular compromise, and appears to elicit useful increases in both systemic vascular resistance and cardiac output (123, 124). Up to two thirds of CDH cases may have biochemical evidence of adrenal insufficiency in the immediate pre and post-operative periods, as observed by Kamath et al. (125). Low cortisol levels were associated with need for higher levels of cardio-respiratory support including ECMO, but not survival.

### Extra-corporeal membrane oxygenation and cardiac function in congenital diaphragmatic hernia

Early ventricular dysfunction is predictive of extra-corporeal membrane oxygenation (ECMO) use in CDH, and ECMO may be an important means of mechanically supporting the failing heart (62). However, there is minimal published data on longitudinal changes of ventricular function during the ECMO therapy. Additionally, decision-making regarding the relative benefits of venoarterial or venovenous ECMO based on the severity of concomitant ventricular dysfunction is at present supported only by clinician experience and opinion and not by published data (126). Bo et al. recently observed that ventricular function improves rapidly over the first days of life on ECMO support and may be monitored using biomarkers including NT-pro BNP (69).

Although cardiac dysfunction is commonly observed before and immediately after commencement of ECMO, it is rarely the cause of failure to wean support. As discussed above, severe LV dysfunction is a transient phenomenon and typically resolves in a matter of days. Nevertheless, ongoing right ventricular dysfunction after ECMO has been described, associated with persistent elevation of pulmonary artery pressure (61).

The two principal phenotypes responsible for ECMO weaning failure are first severe ventilation failure secondary to pulmonary hypoplasia, and second severe ongoing pulmonary hypertension. The latter may be due to irreversible developmental anomalies of pulmonary vasculature structure and function, decruitment of lung, or other factors such as ongoing infection.

Improved understanding of the relative contribution of cardiac dysfunction to individual pathophysiology before and during ECMO may be important for improved ECMO management strategies and help resolve ongoing uncertainties and controversies (127, 128).

### Future priorities in congenital diaphragmatic hernia cardiac function therapy

To achieve effective use of these and other cardiovascular therapies in CDH future priorities should include:

• Standardized hemodynamic assessment parameters in CDH, incorporating routine functional echocardiography. Consensus definitions of pulmonary hypertension and cardiac function and dysfunction based on standardized measures.
• Pathophysiology-based treatment strategie*s* rather than “one-size fits all” approach to use of single agents (1).
• Routine assessment of cardiac function in studies of hemodynamic therapies, as in the recent CoDiNOS trial of IV sildenafil (73).
• Investigation of the relationship between LV performance and response to pulmonary vasodilator therapies.
• Improved understanding of cardiac function to guide individualized ECMO strategies including improved patient selection and timing of repair.
• Investigation of whether improvement in cardiac function translates to improved short and long-term clinical outcomes.

## Surgical repair and cardiac function in congenital diaphragmatic hernia

Current international guidelines recommend delaying CDH repair until physiological stability including normalized blood pressure and lactate have been achieved, but do not specifically reference cardiac function (129). RV function may deteriorate within 72 h of surgical repair (49). In a recent cohort Avitabile et al. observed that RV strain improved in the recovery phase after surgery, though over 50% of cases had ongoing reduction in RV strain (61). Chronic elevation of PVR, exacerbated by surgery, may contribute to this ongoing RV dysfunction. Conversely in the LV, Tanaka et al. have observed improved LV diastolic wall strain following early CDH repair, potentially as a result of removing the compressive action of the hernia (130). Further investigation is required to fully understand the impacts of the herniating abdominal contents, anesthesia and the surgical techniques on cardiac function and hemodynamics in CDH.

Cardiac loading conditions may also be altered in the post-operative period. Chylothorax occurs in 5% of CDH cases after repair or ECMO cannulation, possibly resulting from superior vena cava obstruction (131, 132). The associated reduction in preload may in turn affect ventricular function and cardiac output.

## Long-term cardiac function in congenital diaphragmatic hernia

The natural history of long-term cardiac function in CDH is not well understood.

Kraemer et al. observed that pulmonary hypertension largely resolves in childhood in CDH survivors (133). Similarly, a recent literature review identified highly variable rates of pulmonary hypertension (4.5–38%) in CDH survivors over 2 years old, and diminishing rates by 5 years of age (134). However, the limitations of echocardiographic techniques make accurate non-invasive assessment of pulmonary artery pressure challenging. Ventricular function may be a more sensitive measure of ongoing changes in RV loading conditions and the systemic circulation, as demonstrated in non-CDH pulmonary hypertensive diseases (135–137).

Analysis in our own center demonstrated abnormal RV and LV strain in 70% and 44% of CDH patients respectively at the time of neonatal discharge (Massolo et al., unpublished data). Furthermore, Egan et al. observed abnormal RV strain in CDH survivors at a median of age of 6 years (138). Altered cardiac function, in conjunction with ongoing respiratory compromise, may conceivably contribute to functional exercise restriction in a minority of CDH patients (139).

In survivors of preterm birth there is increasing evidence that altered ventriculo-arterial interactions may contribute to adult cardiovascular dysfunction, raising potential concerns that similar pathologies might occur in CDH (140–142). Prospective, longitudinal studies are now required to understand lifelong patterns and functional significance of cardiac function in CDH.

Cardiac catheterization may be an important adjunct for longitudinal hemodynamic assessment in CDH. Limited case series have demonstrated the ability to directly assess pulmonary artery and intra-cardiac pressures, including in the left heart, ventricular outputs, occult shunts, additional congenital lesions, and responsiveness to therapies including pulmonary vasodilators in CDH (143, 144). Earlier, standardized use of cardiac catheterization in cases of CDH with ongoing hemodynamic compromise, is an important future consideration.

In young adults who have been born prematurely and have low adaptive capacity during exercise cardiac catheterization has revealed evidence of pulmonary hypertension (145). This raises the question of whether catheterization may also be indicated in CDH survivors who have ongoing evidence of cardio-respiratory exercise intolerance (146, 147).

## Discussion

There is increasing evidence and recognition of the heart’s contribution to disease pathophysiology in CDH (68). However, the “knowns” are outweighed by ongoing uncertainties or “unknowns,” as summarized in Table 6. Addressing these may be critical to developing new therapeutic approaches and improved outcomes.

**TABLE 6:** The heart in CDH: Knowns, unknowns and future research priorities.

Fetus	Early postnatal period	Peri-operative period and ECMO	Post discharge, childhood and beyond
**Knowns**			
Fetal cardiac hypoplasia: Preliminary evidence of increase in ratio of right:left sided dimensions in later pregnancy in left-sided CDH.	Potential for RV and/or LV dysfunction in transitional period.	Improvement of cardiac function during ECMO	Preliminary evidence of cardiac dysfunction at discharge and into childhood
	Relationship between early ventricular dysfunction and neonatal outcome	Preliminary evidence of patterns of RV and LV function post op	
**Unknowns/future priorities**			
Mechanisms of ventricular hypoplasia	Mechanisms of postnatal cardiac dysfunction	Contribution of cardiac dysfunction to pathophysiology pre-and during ECMO	Natural history and clinical significance on long-term cardiac dysfunction in CDH survivors
Pre-natal cardiac function	Effect of cardio-tropes on outcome in CDH	Utility of cardiac function assessment to guide therapeutic strategy including timing of CDH repair and ECMO	
Relationship between pre-natal cardiac function, cardiac dimensions	Relationship between pulmonary vasodilators and LV function	Post-natal patterns of RV and LV function and contribution to post-operative morbidity.	
Predictive potential of fetal cardiac dimensions and function	Impact of pathophysiology-based transitional management on cardiac function	Impact of related morbidities on cardiac function including nutrition, infection, gastro-esophageal reflux.	Long-term ventriculo-arterial interactions in CDH survivors
Relationship between fetal cardiac dimensions and function and postnatal cardiac dimensions and function			
Effect of fetal therapies on pre- and post- natal cardiac development and function			
International consensus, standardized assessment tools and definitions of cardiac function and pulmonary hypertension for research and clinical management			
Relationship between cardiac development and function, fetal brain development, postnatal brain injury, and long-term neurological function			
Phenotyping of CDH pathophysiology: relative contributions and frequency of cardiac dysfunction, pulmonary hypertension and pulmonary hypoplasia			
Cellular, genetic and metabolic factors associated with myocardial dysfunction at all ages			

Beginning with the fetus, further investigation is required to investigate the nature and mechanisms of fetal LV hypoplasia at a cellular, metabolic, genetic and morphometric level, as well as the functional significance for fetal hemodynamics including cerebral perfusion. The relationship between fetal cardiac dimensions and function, early postnatal function and outcomes is also a priority that may identify improved prenatal predictors and postnatal therapeutic strategies. Assessment of any fetal therapies should include the impact on the heart, as well as the lungs and pulmonary vasculature.

Though RV and LV function have been characterized in early postnatal life, a deeper physiological understanding of underlying ventricular interdependence, ventriculo-arterial and cardio-respiratory interactions at this time will enable more informed, physiology-based therapeutic approaches.

Use of cardiovascular pharmacotherapies in CDH remains a challenging area. Ongoing trials of milrinone and pulmonary vasodilators may shed some light (73, 113). Hemodynamic assessment should be incorporated in all future interventional studies in CDH. To do so requires urgent consensus on what to measure, how, and when, as well as standardized definitions of cardiac function and pulmonary hypertension. This will enable the robust, multi-center data collection and analysis required in this rare disease.

Investigating new therapeutic paradigms may also be important. Rather than a “one size fits all” approach, improved assessment of individual patient pathophysiological phenotype and the relative contributions of cardiac function, alongside pulmonary hypertension and ventilatory function, may lead to improved, targeted use of therapies including pulmonary vasodilators, cardiotropes, and vasopressors. This approach may also improve ECMO strategies addressing thorny uncertainties around patient selection and timing of repair both on and off ECMO.

The relationship between the heart and the brain in CDH is also a critical area for research, to understand the neuro-cardiovascular coupling mechanisms contributing to fetal brain development and postnatal injury in CDH, and their relationship to long-term neurodevelopment.

Finally, the long-term nature of cardiovascular function in CDH survivors is another priority, to determine whether longer-term changes in cardiac morphology and function, or abnormalities of the systemic or pulmonary circulation, impact functional status later in life.

Addressing each of these uncertainties will require innovative approaches. First, in terms of methodology combining human clinical investigation, animal studies, and novel cellular and organoid models, as well as new imaging modalities including advanced functional echocardiography and MR (148–150). Second, by applying learning from other related conditions, including congenital heart disease and non-CDH pulmonary hypertensive disease. Third, and above all, collaboration is required to share knowledge, consolidate expertise and capabilities between researchers, clinicians and people with CDH themselves.

We hope that this review can act as a call to action, highlighting the importance of the heart and the next steps to progress our understanding, develop new therapies, and improve outcomes in CDH.

## Author contributions

NP, AM, UK, and FK contributed to the concept, outline of the manuscript, reviewed drafts, and approved the final manuscript. NP provided co-ordination of each authors’ contributions. All authors contributed to the article and approved the submitted version.
